# Complete Genome Sequence of an Avian Paramyxovirus Type 4 Strain Isolated from Domestic Duck at a Live Bird Market in South Korea

**DOI:** 10.1128/genomeA.00318-17

**Published:** 2017-05-18

**Authors:** Erdene-Ochir Tseren-Ochir, Seong-Su Yuk, Jung-Hoon Kwon, Jin-Yong Noh, Woo-Tack Hong, Jei-Hyun Jeong, Sol Jeong, Yu-Jin Kim, Kyu-Jik Kim, Ji-Ho Lee, Jun-Beom Kim, Joong-Bok Lee, Seung-Yong Park, In-Soo Choi, Sang-Won Lee, Chang-Seon Song

**Affiliations:** a Avian Diseases Laboratory, College of Veterinary Medicine, Konkuk University, Seoul, Republic of Korea; b Konkuk Ctc bio Animal Vaccine (KCAV) Co., Ltd., Seoul, Republic of Korea

## Abstract

We report here the first full-genome sequence of an avian paramyxovirus type 4 (APMV-4) strain isolated from a domestic mallard duck at a live bird market in South Korea. Phylogenetic analyses provide genetic information on a new genetic clade, APMV-4, isolated from a domestic duck and evidence of APMV-4 exchange between poultry and wild birds.

## GENOME ANNOUNCEMENT

Avian paramyxoviruses (APMVs) are enveloped, negative-sense, single-stranded RNA viruses belonging to the genus *Avulavirus* of the family* Paramyxoviridae* (1–3). To date, 12 different serotypes, APMV-1 to APMV-12, have been recognized (1, 4–6). The APMV-4 serotype has been frequently isolated from wild and domestic waterfowls around the world since 1975, with its first isolation being from a duck in Hong Kong. So far, a total of 7 full-length and 115 partial APMV-4 genome sequences have been reported at GenBank, including one full-length sequence (accession no. EU877976) and 11 partial gene sequences (3, 7–11) of South Korean isolates. Genome analyses suggest that APMV-4 strains are potentially divided into two lineages, the Western Hemisphere and the Eastern Hemisphere (EH) lineages, with genetic distances between and within lineages (11, 12), forming five major clades (13). All of previous South Korean strains were isolated from wild waterfowls and involved the EH lineage within at least two clade genetic subgroups (3, 12).

In this study, we report the first full-genome sequence the APMV-4 strain isolated from tissue sample of an apparently healthy domestic mallard duck (*Anas platyrhynchos*) at a live bird market (LBM) in South Korea. The tissue (cecal tonsil) homogenate was inoculated in 9- to 11-day-old specific-pathogen-free (SPF) chicken embryonated eggs and tested for hemagglutinin activity (HA) with 1% chicken red blood cells (RBC). Viral RNA was extracted using RNeasy minikit (Qiagen), and full-length cDNA was synthesized using the SuperScript III first-strand system for real-time PCR (RT-PCR; Invitrogen), with random hexamers. The cDNA was used to prepare Ion Fragment sequencing libraries, and the complete genomic sequence was determined by next-generation sequencing (NGS), using the Ion Torrent PGM (Life Technologies, Inc.) sequencer platform. The mapping and annotation were performed by Geneious version 8.1.9 (14), with a reference sequence (APMV-4/KR/YJ/06, GenBank accession no. EU877976).

The full genomic sequence of APMV-4/Mallard/LBM/Korea/019/2012 has 94.6% sequence identity with strains from Belgium, 94.3% with strains from South Korea, 93.4% with strains from China, South Africa, and the Russian Federation, 92.6% with strains from Hong Kong, and 85.8% with strain from Delaware. The amino acid sequence identities of the N-P-M-F-HN-L proteins between the South Korean LBM strain and Hong Kong (prototype) strain are 95.2%, 93.7%, 94.8%, 95.6%, 94.6%, and 94.9%, respectively. The fusion protein of the South Korean LBM strain has the same sequence of cleavage site (DIQPR↓F) that all other strains previously reported elsewhere have (10, 12, 13, 15, 16).

Phylogenetic relationships and genetic distances were analyzed by the neighbor-joining method with 1,000 replicates bootstrap values under the Kimura 2-parameter model, using the MEGA 6.0 software (17).

The analysis indicated that the South Korean LBM strain is involved with the EH lineage and is most closely related to one Japanese (accession no. KT732293) wild bird strain but separated from the other five major clades. Thus, the present results provide significant genetic information on a new genetic clade of APMV-4 and evidence of APMV-4 exchange between poultry and wild birds, indicating the further enhanced surveillance of poultry and wild bird populations in South Korea would be helpful for understanding of the global transmission and epidemiology of APMV-4.

### Accession number(s).

The complete genome sequence of the strain APMV-4/Mallard/LBM/KR/019/2012 has been submitted to GenBank under the accession no. KY681684.
